# An Improved Longitudinal Driving Car-Following System Considering the Safe Time Domain Strategy

**DOI:** 10.3390/s24165202

**Published:** 2024-08-11

**Authors:** Xing Xu, Zekun Wu, Yun Zhao

**Affiliations:** School of Information and Electronic Engineering, Zhejiang University of Science and Technology, Hangzhou 310023, China; xuxing3220@163.com (X.X.); w15635453880@163.com (Z.W.)

**Keywords:** intelligent transportation, car-following model, safe time headway, connected vehicle

## Abstract

Car-following models are crucial in adaptive cruise control systems, making them essential for developing intelligent transportation systems. This study investigates the characteristics of high-speed traffic flow by analyzing the relationship between headway distance and dynamic desired distance. Building upon the optimal velocity model theory, this paper proposes a novel traffic car-following computing system in the time domain by incorporating an absolutely safe time headway strategy and a relatively safe time headway strategy to adapt to the dynamic changes in high-speed traffic flow. The interpretable physical law of motion is used to compute and analyze the car-following behavior of the vehicle. Three different types of car-following behaviors are modeled, and the calculation relationship is optimized to reduce the number of parameters required in the model’s adjustment. Furthermore, we improved the calculation of dynamic expected distance in the Intelligent Driver Model (IDM) to better suit actual road traffic conditions. The improved model was then calibrated through simulations that replicated changes in traffic flow. The calibration results demonstrate significant advantages of our new model in improving average traffic flow speed and vehicle speed stability. Compared to the classic car-following model IDM, our proposed model increases road capacity by 8.9%. These findings highlight its potential for widespread application within future intelligent transportation systems. This study optimizes the theoretical framework of car-following models and provides robust technical support for enhancing efficiency within high-speed transportation systems.

## 1. Introduction

Highways are critical transportation networks connecting cities and regions, and their traffic status directly impacts the efficiency of daily commuting, freight logistics, and emergency responses. During peak hours, the traffic conditions of highways often become complex and changeable, and vehicles need to reserve extra time in trip planning to cope with possible delays, affecting traffic efficiency and increasing people’s costs. Many expressways face serious congestion problems, especially near the entrance and exit of cities and significant traffic nodes. Sugiyama et al. [1] revealed through a circular road experiment that the uneven acceleration and deceleration behavior of human drivers can cause traffic jams even in barrier-free environments, the so-called “Phantom Traffic Jams” phenomenon, which is particularly prominent on highways.

With the advancement of science and technology and the emergence of intelligent networked vehicles, new opportunities have arisen to address this issue. Connected and automated vehicles (CAVs) can operate at a higher density and synchronicity while maintaining a safe distance, significantly increasing road capacity. Moreover, CAVs can mitigate traffic accidents caused by human error and alleviate traffic congestion that result from unforeseen events. In existing research, car-following models are primarily categorized into two types: analytical modeling based on kinematics or other physical/mathematical principles and the application of big data and machine learning technology for data mining to learn and simulate human driving styles. The latter encompasses supervised learning, unsupervised learning, and reinforcement learning [2,3,4,5]. Although machine learning-based models can partially replicate human driving behaviors, such imitation is not always effective in resolving highway congestion issues. With the development of intelligent connected vehicles, modern models can leverage more input information in a connected vehicle environment, such as the acceleration of the preceding vehicle. However, many traditional follow models need help integrating these new parameters efficiently. The classic IDM model introduces dynamic expected safe distance as a crucial factor [6]. However, the current calculation method does not take into account the effect of weather conditions on vehicle–ground friction. Furthermore, Ploeg et al. [7] proposed the ACC model for autonomous driving systems to reduce the feasible time interval between the front and rear vehicles [8]. Autonomous driving car-following models like Cooperative Adaptive Cruise Control (CACC) require the adjustment of numerous parameters, which adds complexity to the model [9,10]. Particularly within a specific speed range and distance between front and rear vehicles where speed differences are minimal, these models exhibit limited responsiveness towards changes in distance.

This paper proposes enhancements to the traditional physics-based vehicle behavior model to simulate vehicle behavior in actual traffic environments effectively. Our approach involves utilizing a phased classification of vehicle behavior to analyze and calculate the relationship between vehicles and driving under different traffic conditions, thereby accurately reflecting the dynamic response of vehicle behavior. The primary objective of these improvements is to enhance both efficiency and safety of traffic flow. The specific innovations presented in this study are as follows: (1) While enforced approaching the car-following state, the concept of “absolute safe time headway” and “approaching expected safe distance” based on an optimal velocity model to maximize the driving speed of vehicles is introduced. (2) Proposing the concept of “relative safe time headway” during a progressively far car-following state, along with dynamic deceleration difference according to different road conditions, aiming at ensuring an optimal speed relationship for improved vehicle safety following. (3) Enhancing the IDM model’s dynamic expected distance calculation method by significantly considering weather conditions, making it more suitable for complex weather conditions on actual roads. The remainder of this article is organized as follows: Section 2 introduces relevant cutting-edge studies. Section 3 elaborates on the algorithm used in this study, including design details. Section 4 describes simulation experiments showcasing experimental results compared with existing methods. Finally, Section 5 presents concluding remarks.

## 2. Literature Review

Many models based on kinematics were proposed very early, such as the classic Gipps’ Model [11], General Motors Model [12,13], OVM [14], Krauss Model [15], etc. In the GM model, the parameters γ and n are the core parameters, which determine the sensitivity of the model and how the vehicle reacts; a higher value of γ means that the vehicle reacts faster to changes in the speed of the leading vehicle, but it may also lead to a more unstable system. The critical parameters of the OVM model are α and the optimal velocity function V(∆x). The parameter α can adjust the vehicle’s speed to the optimal speed; higher values allow the vehicle to adjust to the optimal speed faster but may introduce multiple degrees of oscillation or instability. By introducing a negative velocity difference, Helbing et al. [6,16] proposed the GFM model to overcome the unrealistic acceleration and deceleration problem of the OVM model. Jiang et al. [17] considered both positive and negative speed differences and proposed the FVDM model to enhance the ability to simulate the starting process of an automobile platoon. Gong et al. [18] proposed the asymmetric full velocity difference model (AFVDM) to consider the asymmetry of speed differences between vehicles on the road. It can be found that this model includes the study of the car-following behavior of the GFM model and FVDM model. To optimize the traffic congestion problem, Jin et al. [19] proposed introducing lane width into the FVDM model and establishing an improved car-following model. Yu et al. [20], based on the FVDM model, comprehensively considered the relationship between the headway, speed difference, and acceleration, proposing the full velocity difference acceleration model (FVDAM) to suppress traffic congestion and increase traffic capacity. However, in the actual traffic environment, the dynamic speed relationship between vehicles on the road is variable, and a single car-following distance makes it difficult to describe this change effectively. Some scholars have studied the improved CACC model based on queue behavior [21]. In other studies, some scholars have proposed the classic IDM model by introducing factors such as the driver’s driving style and psychology, improving this problem by dynamic expected distance. Moreover, IDM+, an adapted version of IDM, can better focus on traffic flow stability [22]. However, the high complexity of the model increases the computational cost and requires accurate parameter setting. Helly’s model is easy to analyze and simulate through a simple mathematical expression while improving the vehicle’s dynamic response to the preceding vehicle [23,24]. However, the neglect of a driver’s behavior and environmental factors results in the model having limited performance under nonlinear and complex traffic conditions. In recent studies, the connected car-following model has been widely mentioned. Some scholars proposed car-following models based on event-driven and dynamic factors. Similar to the previous traditional models, these models still assume that drivers will respond to changes in relative speed or other factors [25,26,27]. In particular, Ossen et al. [28] assumed different distributions of perception and reaction times for information-assisted and information-unassisted drivers. Jia et al. [29] proposed a psychological and physical-based response to car-following behavior, in which the influence of various perception thresholds, such as the minimum expected car-following distance and the minimum expected stopping distance, on the driver’s response was considered. Xin et al. [30] introduced a variable vehicle spacing strategy into the full speed difference model (FVDM) and proposed two new car-following models, which adopted a constant time spacing headway (CTH) strategy and a variable time spacing headway (VTH) strategy, respectively. Through steady state analysis and numerical simulation, it is found that the variable vehicle spacing strategy with appropriate parameters can help to improve the stability of traffic flow and avoid unstable traffic phenomena. Feng et al. [31] proposed a joint traffic control framework based on DSRC technology to reduce vehicle delay and emissions through signal and trajectory optimization, simplify the objective function, and obtain the analytical solution of the two-stage model.

## 3. Model

In the real traffic environment, the road is usually composed of multiple lanes, but the lane change model is also an important part of the automatic driving system. After changing lanes under different conditions, the vehicle still maintains the local single-lane car-following state. We base our inquiry on the traditional dynamic model, which is limited to the case of insignificant lane changes. Therefore, we only consider a one-way lane in the theoretical analysis part of this paper. The vehicle information obtained at the current time calculates the acceleration at the next time to adjust the vehicle’s driving mode. The optimal speed strategy based on driving safety principles is realized to improve the vehicle travel time and road occupancy rate on highways and urban expressways. With the emergence of intelligent and connected vehicles, the car-following model based on a modern connected vehicle environment will have more abundant input information, such as the acceleration of the leading vehicle. By introducing this information, the traditional car-following model can effectively improve the shortcoming of insufficient information, and it can more accurately and reasonably adjust the car-following behavior of its vehicle.

Gang et al. [32] summarized and subdivided the driving modes in high-speed situations, including 12 modes of free straight driving, approaching, and long-distance following. Based on this research, this paper focuses on the relationship between dynamic expected car-following distance and actual distance. It divides various driving modes from three directions: enforced approach car-following, steady state car-following and progressively far car-following. Other car-following morphologies are combined in the interspersed form. The concepts of “absolute safe time headway” and “relative safe time headway” are proposed by analyzing the relationship between high-speed vehicles’ front and back time intervals. We consider the current input information of the vehicle, including the speed $v_{n}$ and the acceleration $a_{n}$ of the following vehicle, the speed $v_{n+1}$ and the acceleration $a_{n+1}$ of the leading vehicle, the headway distance $S_{\alpha }$, and the dynamic expected safety distance $S^{*}$. The car-following process can be divided into three stages based on the relationship between the distance headway and the expected safety distance. When $S_{\alpha }$ is greater than $S^{*}$, it is expressed as enforced approach car-following. At this stage, the expected behavior of the following vehicle is to control the relative speed of the following vehicle, gradually make the distance between the two vehicles close to the expected safe distance, and adjust the speed to achieve the expected state of car-following. When $S_{\alpha }$ equals $S^{*}$, it is formulated as steady state car-following, and we think an error of ±1 m is allowed. After reaching the ideal car-following state, the following vehicle will continuously follow the leading vehicle at a relatively stable speed and spacing. When $S_{\alpha }$ is less than $S^{*}$, it is expressed as progressively far car-following. In this state, the car-following behavior of the following vehicle is beyond the scope of safe driving, and there is a risk of collision. It is hoped that the desired safe spacing can be restored by adjusting the driving strategy of the following car to increase the car-following distance. With this precondition, the detailed problem of different car-following states continues to be analyzed in depth.

**Figure 1:**
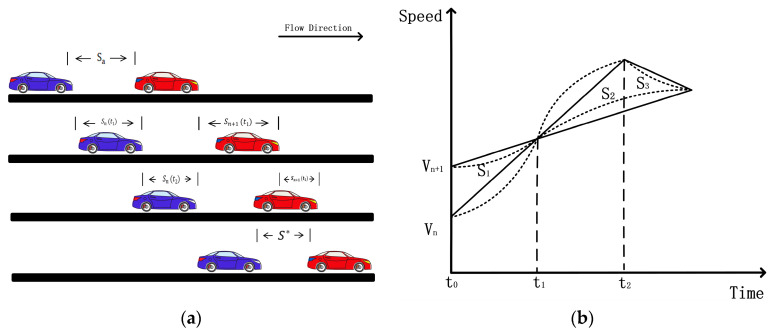
Part (**a**) represents the change of driving relationship between its vehicle n and its leader vehicle n + 1 in the enforced approach car-following phase. The vehicle’s acceleration may be a different nonlinear relationship based on different driving styles. For the convenience of observation, we use the linear function to plot part (**b**).

As shown in Figure 1a, under the maximum acceleration limit in the enforced approach car-following phase, the following vehicle needs to adjust its driving strategy to chase the leading vehicle to reduce the distance between them. The acceleration of the following vehicle must be greater than that of the leading vehicle during this period. However, at any time t, there are three different speed relations. Figure 1b is drawn based on the case when the following vehicle’s velocity is smaller than the leading vehicle’s. As can be seen in figure, the traveling relation $v_{n}>v_{n+1}$ can be found after moment $t_{1}$. So, we start from time $t_{0}$ to explore the driving strategy of the following car at that stage. Since the speed of the leading vehicle at time $t_{0}$ is greater than that of the following vehicle, $S_{1}$ represents the increased distance between the two vehicles from time $t_{0}$ to time $t_{1}$, and $S_{2}$ represents the decreased distance between the two vehicles from time $t_{0}$ to time $t_{1}$. In this case, the distance between the two vehicles at time $t_{2}$ only returns to the distance relationship at the initial time $t_{0}$. From this, it is known that there is a free acceleration process for the following vehicle before time $t_{1}$ when the speed relationship changes, which conforms to the safe driving strategy. So critical time $t_{1}$ is described as the “absolute safety time headway”. In this way, the following car has a specific time range, and in the car-following mode, the accessible straight driving mode is realized. This critical time $t_{1}$ is calculated as follows:

(1) $$\begin{array}{c} S_{n}=v_{n}t+\frac{1}{2}a_{n}t^{2} \end{array}$$

(2) $$\begin{array}{c} S_{n+1}=v_{n+1}t+\frac{1}{2}a_{n+1}t^{2} \end{array}$$

when $t=t_{1}$, we hope $v_{n}=v_{n+1}$; so, $v_{n}+a_{n}t=v_{n+1}+a_{n+1}t$.

(3) $$\begin{array}{c} t=\frac{v_{n+1}-v_{n}}{a_{n}-a_{n+1}}or\frac{v_{n}-v_{n+1}}{a_{n+1}-a_{n}} \end{array}$$

where time t is called the “absolute safety time headway”, during which, based on the principle of the most velocity model, we adjust the acceleration of the following vehicle to the maximum acceleration $a^{\alpha }$. When the time scale reaches $t_{2}$, the following vehicle starts to chase to reduce the distance between the two vehicles. At this time, the driving demand of the following vehicle is to approach the headway distance to the desired safe vehicle distance while adjusting the speed to achieve the desired following state. The formula is derived as follows:

(4) $$\begin{array}{c} S=S_{\alpha }-S^{*}=S_{n}-S_{n+1} \end{array}$$

(5) $$\begin{array}{c} S=v_{n}t+\frac{1}{2}a_{n}t^{2}-v_{n+1}t-\frac{1}{2}a_{n+1}t^{2} \end{array}$$

(6) $$\begin{array}{c} v_{n}+a_{n}t=v_{n+1}+a_{n+1}t \end{array}$$

(7) $$\begin{array}{c} t=\frac{v_{n}-v_{n+1}}{a_{n+1}-a_{n}} \end{array}$$

(8) $$\begin{array}{c} 2S=\frac{{\left(v_{n}-v_{n+1}\right)}^{2}}{\left(a_{n+1}-a_{n}\right)} \end{array}$$

(9) $$\begin{array}{c} a_{n}=a_{n+1}-\frac{{\left(v_{n}-v_{n+1}\right)}^{2}}{2\left(S_{\alpha }-S^{*}\right)} \end{array}$$

thus, we obtain the adjustment formula in the enforced approach car-following phase as

(10) $$\begin{array}{c} a\left(t+T\right)=\left\{\begin{array}{c} a^{\alpha },v_{n}\leq v_{n+1}\\ a_{n+1}-\frac{∆v^{2}}{2\left(S_{\alpha }-S^{*}\right)},v_{n}>v_{n+1} \end{array}\right. \end{array}$$

where T denotes the time step in the simulation.

In the steady state car-following phase, the ideal state is when the following movements of the rear and front vehicles are synchronized as if they are connected as carriages. However, this is not feasible because each vehicle is individual. Therefore, we try to simulate this ideal state as much as possible and design the following formula:

(11) $$\begin{array}{c} a\left(t+T\right)=\frac{1}{1+e^{-\left(\frac{a_{n+1}}{a_{n}}\right)}}a_{n+1} \end{array}$$

where the rear vehicle’s speed adjustment is based on responding to changes in the front vehicle’s acceleration and maintains stable following behavior by smoothly picking up the front vehicle’s changing trend.

**Figure 2:**
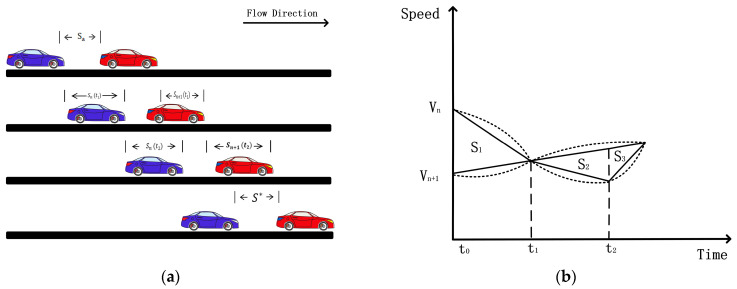
Part (**a**) represents the variation of the driving relationship between the own vehicle n and the leader vehicle *n* + 1 in the progressively far car-following phase. Based on different driving styles, the vehicle’s acceleration may be a different nonlinear relationship, and for easy observation, we use a linear function to graph (**b**).

Both safety hazards and road congestion must be considered in the progressively far car-following phase. If the conventional deceleration speed cannot match the speeds within a safe distance, it may cause a safety hazard. Therefore, the deceleration speed needs to be adjusted to ensure that the speeds of the two vehicles can be safely matched while increasing the distance. Maximizing the deceleration speed may lead to sudden deceleration, which may cause a chain reaction and road congestion for the following vehicles. Therefore, choosing an appropriate deceleration speed and adjustment strategy is crucial to balance safety and smoothness. Based on the safe driving rules, the following vehicle needs to adjust its driving strategy to get out of the following area with safety hazards and restore the desired safe driving distance. As mentioned above, there are three different initial speed relations at any moment. As shown in Figure 2b, the driving relationship of $v_{n}<v_{n+1}$ can be found after moment $t_{1}$. In order to reduce the formulation of equations, we analogize the case of $v_{n}<v_{n+1}$ to the enforced approach car-following state, which approximates the case of Equations (4)–(9), and we do not provide an additional description. However, the situation in the progressively far car-following phase, where the speed of the rear vehicle is greater than that of the front vehicle, is very tricky. Suppose we make the headspace approach the desired safety distance while still requiring the rear vehicle speed to reach the desired following state. Due to the regular deceleration mode, it is easy to cause insufficient deceleration distance, resulting in a potential safety hazard of a collision. The use of maximum deceleration will lead to the formation of road congestion. So, in this case, we propose to set the dynamic speed reduction difference according to different road conditions. The specific formula is derived as follows:

Similar to the above $t_{1}\to t_{2}$ distance change relationship, as shown in Figure 2, we would like the rear vehicle to travel more distance $S_{1}$ than the front vehicle to comply with the safe driving rules. That is, the headway is indented by a certain distance to maintain at least a specific minimum spacing:

(12) $$\begin{array}{c} S_{n}-S_{n+1}\leq S_{\alpha }-S_{min}, \end{array}$$

combining Equations (1) and (2) gives us the following:

(13) $$\begin{array}{c} v_{n}t+\frac{1}{2}a_{n}t^{2}-v_{n+1}t-\frac{1}{2}a_{n+1}t^{2}\leq S_{\alpha }-S_{min} \end{array}$$

Based on the mathematical quadratic function image analysis within Equation (13), the following can be deduced:

(14) $$\begin{array}{c} \mathrm{Let}:\;y=\frac{1}{2}(a_{n}-a_{n+1})t^{2}+\left(v_{n}-v_{n+1}\right)t-\left(S_{\alpha }-S_{min}\right)\leq 0 \end{array}$$

where $S_{min}$ denotes the minimum safe distance, which usually simplifies Equation (14) in the following way:

(15) $$\begin{array}{c} y=\frac{1}{2}At^{2}+Vt-S \end{array}$$

where $A=a_{n}-a_{n+1}\;\mathrm{and}\;A<0;\;V=v_{n}-v_{n+1}\;\mathrm{and}\;V>0;\;S=S_{\alpha }-S_{min}\;\mathrm{and}\;S>0$.

Comparative analysis reveals the time relationship between Equation (15) and Figure 2b. In the time range from $t_{0}$ to $t_{1}$, since the initial speed of its vehicle is more significant than that of the leader vehicle, its vehicle will keep approaching the leader vehicle. Therefore, at the time $t_{1}$, a relatively safe headway time distance can be determined to ensure that the own vehicle can always maintain a minimum safe distance from the leader vehicle during the strategy adjustment process. This critical time is called the “relative safe time headway”.

**Figure 3:**
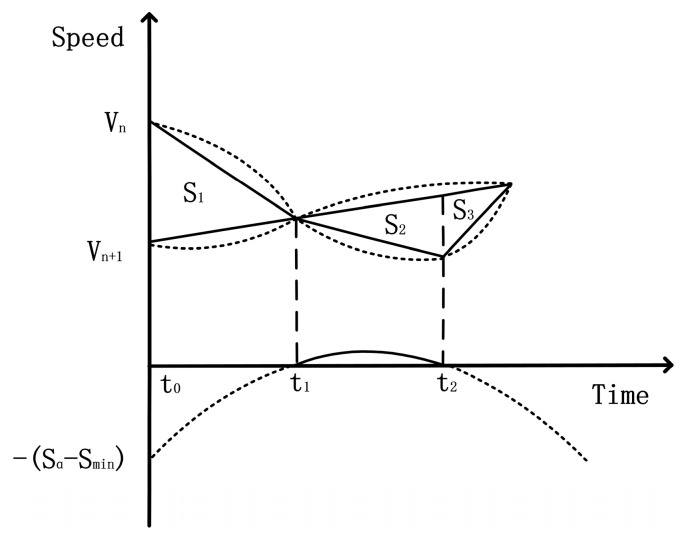
The quadratic function image corresponds to the time relationship between the vehicle’s following process.

The analysis of Figure 2 and Figure 3 and Equation (15) shows that if the numerical relationship under the root sign does not comply with the laws of mathematics, it still does not comply with the laws of motion of physics under the same conditions. Collision hazards will occur, which requires unique emergency braking labels, while in the case of compliance with the conditions, the following conclusions can be drawn:

(16) $$\begin{array}{c} t_{1}=\frac{-V+\sqrt{V^{2}+2AS}}{A}\;\mathrm{and}\;t_{2}=\frac{-V-\sqrt{V^{2}+2AS}}{A} \end{array}$$

where $t_{1}$ is called the relative safety time headway. If the braking time exceeds this range, it will still cause a safety hazard. Based on this moment, we can derive the following:

(17) $$\begin{array}{c} A=\frac{2\left(S-Vt_{1}\right)}{{t_{1}}^{2}} \end{array}$$

here, we get a reasonable strategy for adjusting to the rear car’s fading follow-through:

(18) $$\begin{array}{c} a_{n}=a_{n+1}+\frac{\left(S_{\alpha }-S_{min}\right)-\left(v_{n}-v_{n+1}\right)t}{\tau t^{2}} \end{array}$$

here, the value of τ is 0.5 and this parameter is introduced to eliminate the constant in the equation. Therefore, the adjustment formula for the acceleration in the progressively far car-following phase is

(19) $$\begin{array}{c} a\left(t+T\right)=\left\{\begin{array}{c} a_{n+1}-\frac{∆v^{2}}{2\left(S_{\alpha }-S^{*}\right)},v_{n}<v_{n+1}\\ a_{n+1}+\frac{\left(S_{\alpha }-S_{min}\right)-∆vt}{\tau t^{2}},v_{n}\geq v_{n+1} \end{array}\right. \end{array}$$

In order to synthesize the proposed computational expressions for different states, we introduce symbolic functions to unify the above expressions, and the results are as follows:

(20) $$a\left(t+T\right)=\left\{\begin{array}{l} a^{\alpha }+\left(a_{n+1}-\frac{∆v^{2}}{2\left(S_{\alpha }-S^{*}\right)}-a^{\alpha }\right)\cdot \max\left(0,\;sign\left(∆v\right)\right)\;\;\;\;\;\;\;\;\;\;\;\;\;\;\;\;\;\;\;\;\;\;\;\;\;\;\;\;\;\;\;\;\;\;\;\;\;\;\;\;\;\;\;\;\;\;\;\;\;\;\;\;\;\;\;\;\;\;\;\;\;,S_{\alpha }>S^{*}\\ \frac{1}{1+e^{-\left(\frac{a_{n+1}}{a_{n}}\right)}}∆a_{n+1}\;\;\;\;\;\;\;\;\;\;\;\;\;\;\;\;\;\;\;\;\;\;\;\;\;\;\;\;\;\;\;\;\;\;\;\;\;\;\;\;\;\;\;\;\;\;\;\;\;\;\;\;\;\;\;\;\;\;\;\;\;\;\;\;\;\;\;\;\;\;\;\;\;\;\;\;\;\;\;\;\;\;\;\;\;\;\;\;\;\;\;\;\;\;\;\;\;\;\;\;\;\;\;\;\;\;\;\;\;\;\;\;\;\;\;\;\;\;,S_{\alpha }=S^{*}\\ a_{n+1}+\frac{\left(S_{\alpha }-S_{min}\right)-∆vt}{\tau t^{2}}-\left(\frac{∆v^{2}}{2\left(S_{\alpha }-S^{*}\right)}+\frac{\left(S_{\alpha }-S_{min}\right)-∆vt}{\tau t^{2}}\right)\cdot \max\left(0,\;sign\left(-∆v\right)\right),S_{\alpha }<S^{*} \end{array}\right.$$

In addition, weather conditions significantly affect vehicle driving safety and following behavior. Specifically, different weather conditions change the friction coefficient of the road surface, which affects the braking distance and maneuverability of the vehicle. In rainy or waterlogged roads, the road surface becomes slippery, the friction coefficient decreases, and the vehicle braking distance increases significantly. If this factor is not considered, the vehicle may not be able to slow down or stop in time in an emergency, leading to rear-end accidents. The road surface friction coefficient is further reduced in snow and ice, and the vehicle has a longer braking distance and much weaker maneuverability. It is challenging to ensure the safety of driving in this situation with the traditional following model, and the calculation of the dynamic expected distance in the IDM model lacks this part of the resistance factor, which makes it challenging to realize the demand of the complex environment of the actual road scenarios. In this paper, we consider introducing the friction changes between the road and the vehicle under different weather conditions, and the model can calculate the desired distance more accurately to improve driving safety and stability under complex weather conditions. So, we adjust the influence of different weather conditions on the friction coefficient between the vehicle and the ground as a controllable parameter to more realistically reflect the dynamic desired distance under complex road conditions. The value of ω is determined based on the road smoothness, and the value of ω is adjusted to 1, 0.9, 0.8 and 0.7, respectively, according to different types of dry and wet subgrades (dry, moderately wet, wet and over-wet). Through this setting, the expected car-following distance of the vehicle can be increased in the wet section to improve the safety of car-following. The parameters a and b represent the acceleration and deceleration of the vehicle, and the parameters range from 1$\;m/s^{2}$ to 2.6 $m/s^{2}$ and from 4 $m/s^{2}$ to 4.5 $m/s^{2}$ for different models in the simulation. The sedan parameters used in this paper are 2.6 $m/s^{2}$ and 4.5 $m/s^{2}$. The improved formula for the desired safety distance is as follows:

(21) $$\begin{array}{c} S^{*}=\frac{S_{0}}{\omega }+Tv_{n}+\frac{v_{n}∆v}{2\sqrt{\omega ab}} \end{array}$$

We design an algorithm for interpreting the order of passing to the car-following strategy (see Algorithm 1).
**Algorithm 1****Input:** All information parameters at the current time **Output:** The acceleration $a\left(t+T\right)$ at the next moment under the corresponding adjustment strategy and the adjusted speed information of all CAVs1:**if** there is a leading vehicle ahead, the following condition is formed **then** 2:add all the parameters[a_n, v_n, a_n+1, v_n+1, s*, s, s_main] 3:**for** each $S_{\alpha }>S^{*}$ **do** 4:**if** $v_{n}\leq v_{n+1}$ **then** 5:Set acceleration to $a_{max}$ 6:**else** 7:Set acceleration using Equation (9) 8:**end if** 9:**end for** 10:**for** each $S_{\alpha }=S^{*}$ **do** 11:Set acceleration using Equation (11) 12:**end for** 13:**for** each $S_{\alpha }<S^{*}$ **do** 14:**if**
$v_{n}\geq v_{n+1}$ **then** 15:**if** the relationship between the current velocity difference and the acceleration difference does not meet the requirements of relative safe time headway **then** 16:Set acceleration to a relatively safe set range 17:**else** 18:Set acceleration using Equation (18) 19:**end if** 20:**else** 21:Set acceleration using Equation (11) 22:**end if** 23:**end if** 24:**for** the value of acceleration does not correspond to the real situation **do** 25:Set acceleration to reasonable range 26:**end for** 27:**for** each vehicle **do** 28:Set vehicle speed with increased acceleration information to conform to the road speed limit and the vehicle speed limit 29:**end for**

## 4. Simulation Experiment and Discussion

### 4.1. Basic Simulation

**Figure 4:**
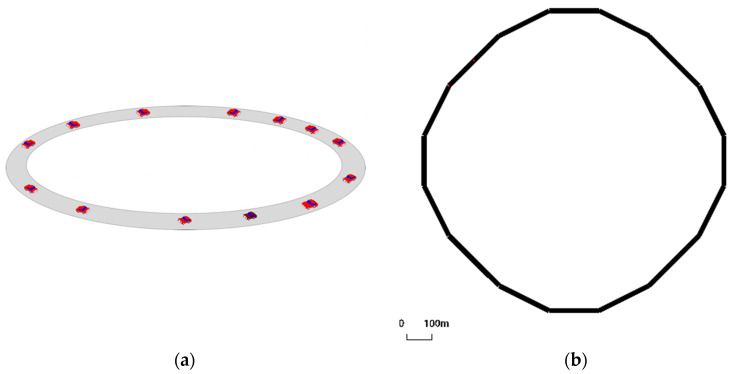
The left figure (**a**) is based on the circular actual road experiment of Sugiyama et al. [1]. The right figure (**b**) shows a simulated road setup using figure (**a**) as a reference.

We simulate a ring road in the Sumo toolkit to investigate the “Phantom Traffic Jams” problem of single-lane expressways. In this paper, we set up a 12,000 m long ring road using the experimental road of Yuki et al. as a benchmark (Figure 4). We set up 12 departure points, with a 1000 m separation between two departure points. Ensure that all vehicles have departed before the starting vehicle travels to the next starting point to avoid an original collision, and finally, make all vehicles travel uniformly on the ring road to ensure the feasibility of the experiment. The time step used in the simulations is 1s. Including the other experiments in the following, we use a uniform parameter criterion for all models. For example, the maximum acceleration is set to 2.6 $m/s^{2}$, the deceleration is 4.5 $m/s^{2}$, and the maximum deceleration during emergency braking is 9 m/s^2^. For comparison, we chose some modified default models in SUMO, such as the modified Krauß model [12], i.e., the Krauss model; the modified CACC model [33,34,35,36]; the improved IDM model of Treiber et al. [37]; and the car-following model Dabiel1 by Daniel Krajzewicz. 

**Figure 5:**
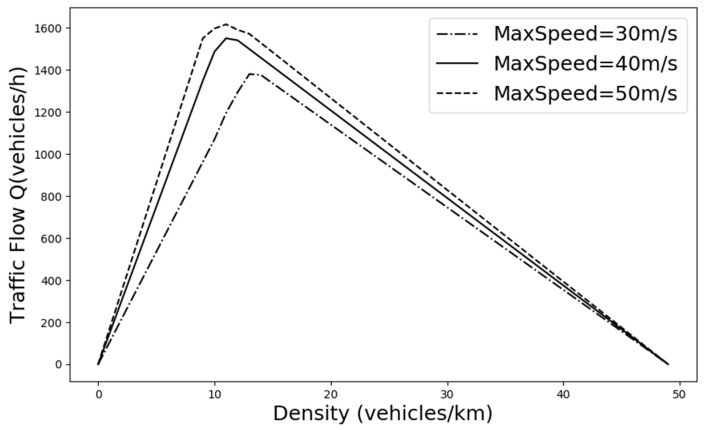
The traffic flow fundamental diagram for this traffic scenario was drawn based on the IDM model to analyze the range of densities suitable for conducting experimental investigations.

**Figure 6:**
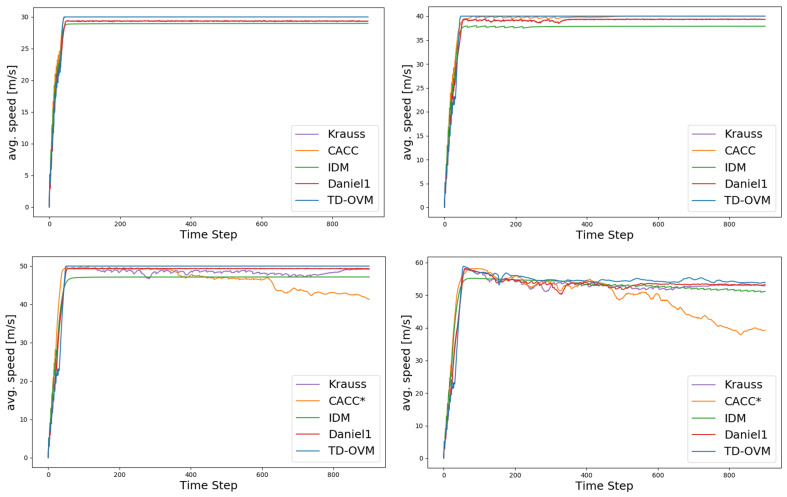
Comparison plot of average speeds across the whole area for each model, with a vehicle density ρ of 11 vehicles per kilometer and maximum road speed limits of 30, 40, 50, and 60. Here, * indicates the presence of a vehicle collision, and the specific collision parameters and road capacity are shown in the table.

**Figure 7:**
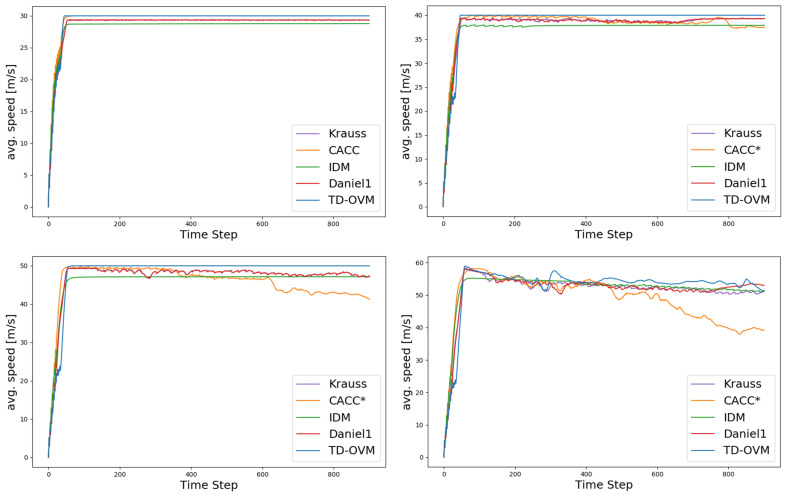
Comparison plot of average speeds across the whole area for each model, with a vehicle density ρ of 12 vehicles per kilometer and maximum road speed limits of 30, 40, 50, and 60. Here, * indicates the presence of a vehicle collision, and the specific collision parameters and road capacity are shown in the table.

As shown in Figure 5, we plot the traffic fundamental diagram based on the IDM model [37] and obtain the bottleneck traffic density of 12 vehicles per km for the simulation experiment scenario. The default values of SUMO were used in the experiments for the parameter settings of the IDM model. The minimum safe distance $S_{0}$ was 2.5 m, the value of the time headway T was 1 s, and the acceleration exponent δ was 4. Figure 6 and Figure 7 demonstrate the comparison of the driving conditions of the vehicles controlled by each model under different traffic density states.

The experimental data in Table 1 and Table 2 show that the traditional model IDM makes it challenging to achieve an effective balance between travel speed and road capacity and is unable to take both into account at the same time, usually giving priority to ensuring adequate vehicle travel speed at the expense of road capacity. This limitation is pronounced on highways and urban expressways, resulting in the inability to utilize these roads’ travel speeds and capacities fully. In complex road environments, it is difficult for CACC models to ensure vehicle driving safety, especially when dealing with traffic conditions with higher traffic density, and these models can hardly ensure both driving speed and safety at the same time. The TD-OVM model performs stably in all traffic environments in this experiment, especially since the speed change curves are smoother and the speed change has been optimized to a certain extent. The experimental results show that the improved TD-OVM model exhibits higher stability and reliability when dealing with complex road environments. It not only improves the traveling speed and safety of the vehicle but also enhances the overall efficiency of the road.

**Figure 8:**
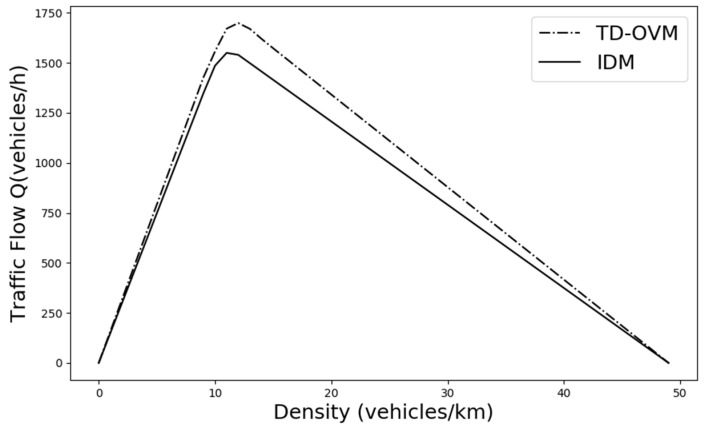
Comparison of the fundamental diagram of the improved model TD-OVM and the basic model IDM, based on a speed limit of 40 m/s.

Finally, the overall performance of the proposed TD-OVM model on the road is summarized. Based on the experimental 40 m/s maximum speed limit case, the flow relationship under different traffic density conditions is obtained. The traffic fundamental diagram of the model is plotted, which is visually compared with the fundamental diagram of the previous IDM model. As shown in Figure 8, compared with the classical IDM model, the improved model has an 8.9% increase in road capacity based on effectively solving the traffic congestion problem, which improves the speed of vehicular traffic and road utilization.

### 4.2. Highway and Urban Expressway Simulations

**Figure 9:**
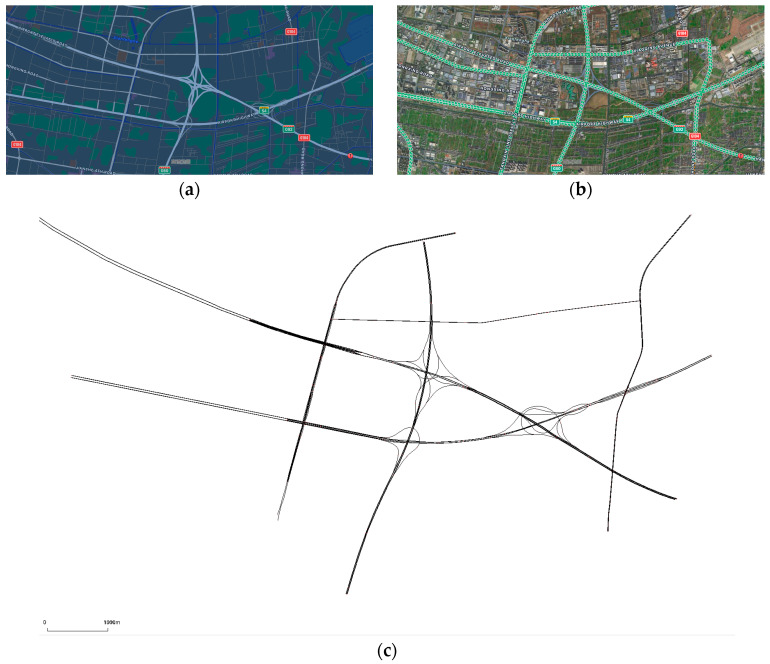
(**a**) City maps. (**b**) Marked map of highways and urban expressways. (**c**) Simulated road trajectory map, which was pruned to remove ordinary urban roads and retain only highways and urban expressways.

To verify the model’s effectiveness in an actual road environment rather than simply simulating a ring road, we excerpt some highways and urban expressways in Xiaoshan District, Hangzhou, for the simulation experiment. The east is to the S4 Airport Highway and the G92 Hangzhou Bay Ring Highway, the south is to the G60 Shanghai-Kunming Expressway, the west is to the Airport Elevated Road and the S4 Airport Highway, and the north is to the Red 15 Line, as shown in Figure 9.

**Figure 10:**
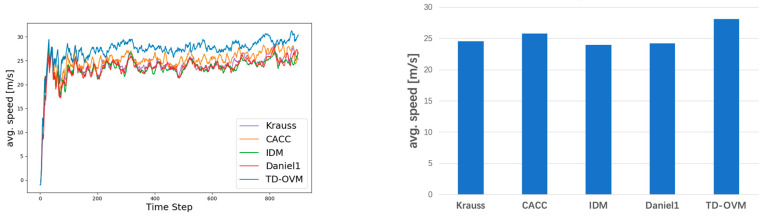
Comparison of average vehicle speeds for roadway volumes at 1000 Vehs/h. The graph on the left shows the trend of average road speeds. The figure on the right shows the average value in the steady state (i.e., after 200 steps).

**Figure 11:**
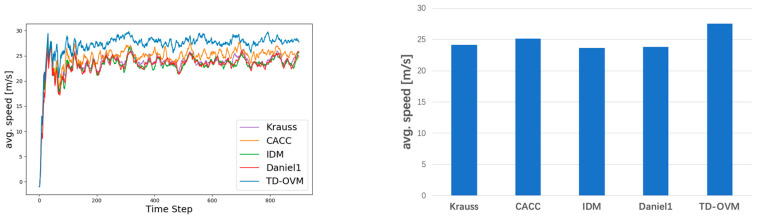
Comparison of average vehicle speeds for roadway volumes at 1400 Vehs/h. The graph on the left shows the trend of average road speeds. The figure on the right shows the average value in the steady state (i.e., after 200 steps).

In this simulation experiment, we simulated road sections containing freeways and urban expressways to fit our research intention. Different traffic densities were set to validate the model’s performance. Specifically, we designed a variety of traffic density scenarios, ranging from low to high, to evaluate the model’s adaptability and stability under various traffic conditions. In each scenario, we recorded key parameters such as vehicle travel speed, headway, acceleration, etc., and analyzed the performance of different models under different traffic densities. Figure 10 and Figure 11 show the comparison of lane average speed under different traffic densities.

**Figure 12:**
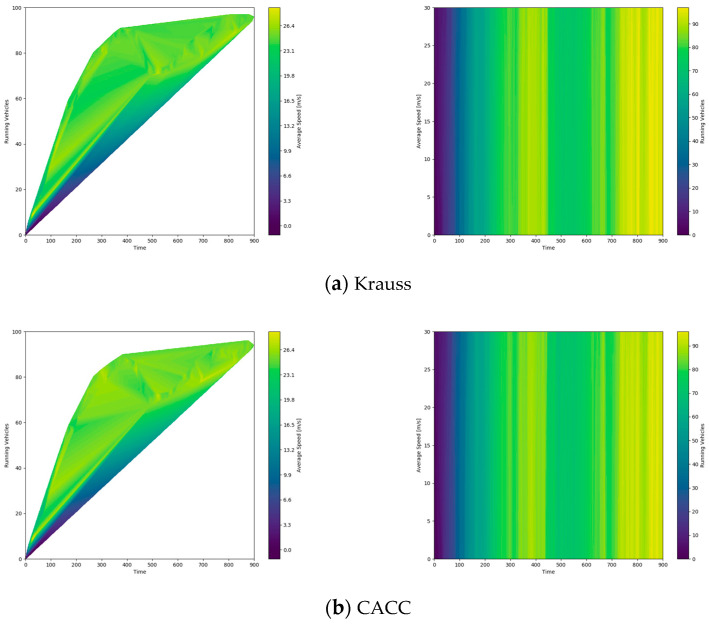

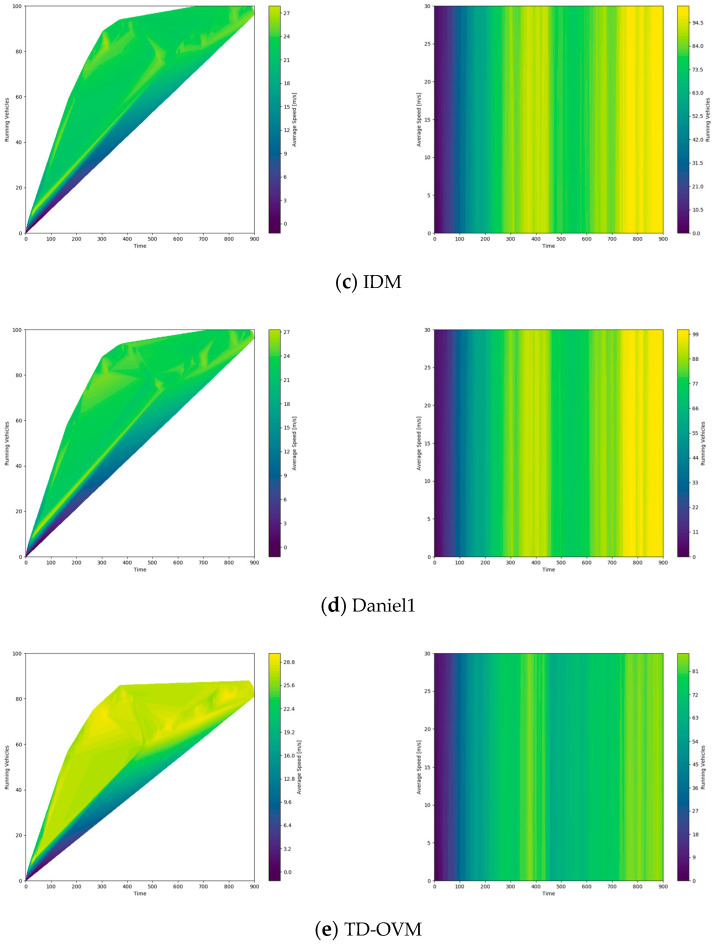
Heat map of average vehicle speed versus stranded vehicle flow on the road for a given time. A higher brightness in the left image indicates a higher average speed on the road, while a lower brightness in the right image indicates that fewer vehicles are stranded on the road, that is, more vehicles have reached their destination or left the road.

As shown in Figure 12, we generated a heat map to show the relationship between the average speed of different models and the volume of vehicles being driven on the road for a certain time at a density of 1400 vehicles per hour. This heat map helps visualize different models’ performance under high-density traffic conditions. In this case, the vehicles in motion data represent the remaining vehicles that have not yet reached the end of the trip, i.e., stranded vehicles.

The above results clearly show that the TD-OVM model has significant advantages over the traditional model in modeling the following behavior of intelligent networked vehicles. By introducing the concept of the interaction between “absolute safe time headway” and “approaching desired distance” and dynamically adjusting the friction coefficient and other improvement measures, the vehicle’s speed is effectively improved under high-density traffic conditions. Compared with the traditional model, the TD-OVM model can adjust the speed more efficiently and realize a faster passing speed while maintaining a safe distance. In addition, the TD-OVM model demonstrates better road accommodation in the simulation experiments. By optimizing the vehicle following strategy and dynamically adjusting the desired distance, the model can effectively reduce the number of stranded vehicles under high-density traffic, thus improving the overall road capacity. While traditional models often lead to congestion under high-density traffic conditions, the TD-OVM model significantly alleviates this problem.

### 4.3. Urban Road Simulation

**Figure 13:**
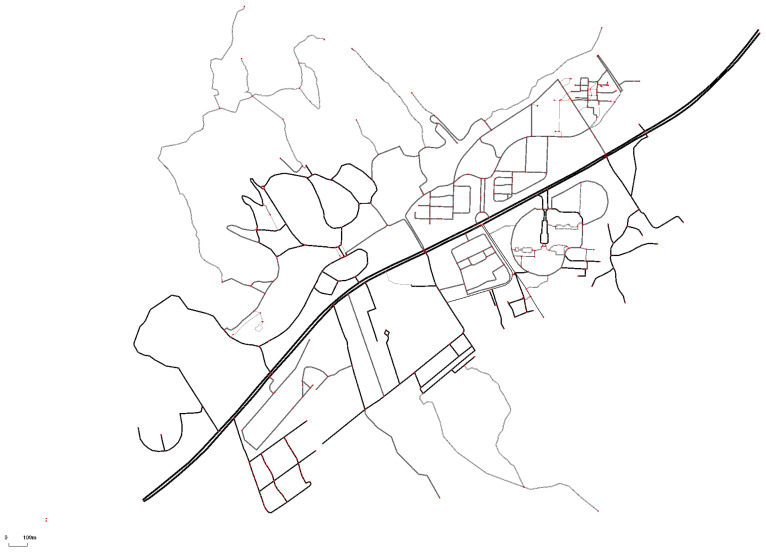
Simulated road network of ordinary urban roads.

Usually, highways have lower road complexity and lack more connectivity between roads. In the urban environment, there is more connectivity information within the road network. To verify the robustness of the model, we added urban road simulation experiments. Although there is no significant difference from the highway in terms of local lanes, the connectivity between roads becomes more complex. The connectivity between roads increases the driving difficulty. Also, the starting and stopping of vehicles increases the complexity of the road network. In order to test the effectiveness of the algorithms in a more prosperous urban road environment, we selected part of the urban roads on the street of Liuxia, West Lake District, Hangzhou, for the simulation experiments (Figure 13). The area contains various road types adapted to the urban traffic environment, such as straight roads, curves, and intersections. This was carried out to more comprehensively evaluate the adaptability and performance of the TD-OVM model in actual traffic environments.

**Figure 14:**
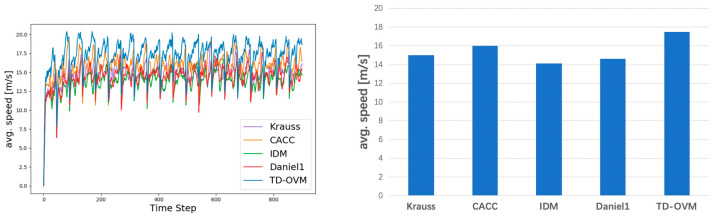
Comparison of average driving speed of different models on urban roads. The graph on the left shows the trend of average road speeds. The figure on the right shows the average value in the steady state (i.e., after 200 steps).

The experimental results show that the TD-OVM model significantly improves the average driving speed, optimizes the headway spacing management, reduces road congestion, and ensures driving safety under high-density traffic conditions (Figure 14). These advantages indicate that the TD-OVM model can better manage headway spacing in complex road environments, avoiding the risk of tailgating caused by too small distances. Meanwhile, it effectively improves driving speed in the following state while maintaining a safe distance between vehicles. It has a wide range of application prospects in modeling the following behavior of intelligent networked vehicles and can effectively improve the efficiency and safety of modern transportation systems.

## 5. Conclusions

This paper proposes an improved car-following model, considering a safe time domain strategy based on analyzing vehicle relationships before and after different phases. It describes the following behavior of vehicles more comprehensively through a specific computational scheme. The aim is to improve the vehicle speed and road capacity and, more realistically, restore the following behavior under complex road and weather conditions. Through an in-depth analysis of the traditional following model, this paper proposes several vital improvements to address the challenges faced by the traditional model in the intelligent connected vehicle environment. In the enforced approach car-following phase, the following vehicle gradually approaches the front vehicle by adjusting its speed to ensure the distance between the vehicles is within a safe range. The model introduces the concept of “absolute safe time headway”, i.e., the following vehicle can accelerate and reduce the distance within a specific critical time to maintain a safe driving strategy. In the steady state car-following phase, the rear vehicle continues to follow the front vehicle with a stable speed and spacing to simulate the ideal synchronized following state. Although complete synchronization is not feasible, the model tries to get as close as possible to this ideal state through fine speed adjustment. In the progressively far car-following phase, the rear vehicle must adjust its deceleration and increase the distance to ensure safety. In this process, dynamic deceleration difference is proposed and adjusted according to road conditions to balance safety and smoothness. These improvements substantially enhance the efficiency and safety of the vehicle. Secondly, this paper improves the classical IDM model by adding the effect of weather conditions on the friction coefficient between the vehicle and the ground. Weather conditions like rain, snow, snow, and ice can significantly change the road surface’s friction coefficient and affect the vehicle’s braking distance and maneuverability. By introducing these factors into the model, TD-OVM can calculate the desired distance more accurately and improve driving safety and stability under complex weather conditions.

In order to verify the effectiveness of the improved model, this paper tests it in various aspects in simulation experiments. The experimental results show that the TD-OVM model exhibits significant advantages under different traffic densities and complex road environments. The model can effectively improve the vehicle’s driving speed by following the behavior while increasing the road capacity, and the traditional traffic congestion problem is significantly improved. In addition, the performance of the TD-OVM model is also excellent in the actual road environment. The adaptability and robustness of the model in actual traffic environments are verified by conducting simulation experiments on some urban roads. The results show that the TD-OVM model can perform well in complex urban road environments, fully demonstrating its potential to improve traffic efficiency and driving safety. Overall, the TD-OVM model proposes a more refined and flexible following strategy by comprehensively considering the various stages of following behavior and external environmental factors, which effectively enhances the safety and efficiency of vehicle driving and significantly improves the modeling accuracy and practicality of vehicle following behavior. Its application in the intelligent networked vehicle environment is promising, and it provides essential theoretical support and a practical foundation for optimizing future transportation systems.

## Figures and Tables

**Table 1:** Comparison of collision scenarios and road capacity for different models with a traffic density ρ of 11 vehicles per kilometer.

Speed (m/s)	Argument	Krauss	CACC	IDM	Daniel1	TD-OVM
30	Collisions	0	0	0	0	0
	Carrying capacity	132/132	132/132	132/132	132/132	132/132
40	Collisions	0	0	0	0	0
	Carrying capacity	132/132	132/132	128/132	132/132	132/132
50	Collisions	0	3	0	0	0
	Carrying capacity	132/132	124/132	109/132	109/132	132/132
60	Collisions	0	13	0	0	0
	Carrying capacity	132/132	126/132	126/132	132/132	132/132

**Table 2:** Comparison of collision scenarios and road capacity for different models with a traffic density ρ of 12 vehicles per kilometer.

Speed (m/s)	Argument	Krauss	CACC	IDM	Daniel1	TD-OVM
30	Collisions	0	0	0	0	0
	Carrying capacity	144/144	144/144	144/144	144/144	144/144
40	Collisions	0	3	0	0	0
	Carrying capacity	144/144	144/144	128/144	144/144	144/144
50	Collisions	0	3	0	0	0
	Carrying capacity	138/144	124/144	109/144	109/144	144/144
60	Collisions	0	13	0	0	0
	Carrying capacity	144/144	126/144	126/144	144/144	144/144

## Data Availability

Data were described in detail in the experimental setup and can be made available upon request.
